# Ultrastructural and dynamic studies of the endosomal compartment in Down syndrome

**DOI:** 10.1186/s40478-020-00956-z

**Published:** 2020-06-24

**Authors:** Alexandra Botté, Jeanne Lainé, Laura Xicota, Xavier Heiligenstein, Gaëlle Fontaine, Amal Kasri, Isabelle Rivals, Pollyanna Goh, Orestis Faklaris, Jack-Christophe Cossec, Etienne Morel, Anne-Sophie Rebillat, Dean Nizetic, Graça Raposo, Marie-Claude Potier

**Affiliations:** 1 Paris Brain Institute (ICM), CNRS UMR7225, INSERM U1127, Sorbonne Université, Hôpital de la Pitié-Salpêtrière, Paris, France; 2 Sorbonne Université, Département de Physiologie, Hôpital de la Pitié-Salpêtrière, Paris, France; 3 CryoCapCell, 155 Bd de l’hôpital, 75013 Paris, France; 4 Institut Curie, PSL Research University, CNRS, UMR144, Structure and Membrane Compartments, Paris, France; 5 Equipe de Statistique Appliquée, ESPCI Paris, PSL Research University, UMRS 1158, Paris, France; 6 The Blizard Institute, Barts and the London School of Medicine and Dentistry, Queen Mary, University of London, London, UK; 7 ImagoSeine Imaging Core Facility, Institut Jacques Monod, CNRS UMR7592, Université Paris-Diderot, Sorbonne Paris Cité, Paris, France; 8 Institut Necker-Enfants Malades (INEM), INSERM U1151 CNRS UMR 8253, Université Paris Descartes-Sorbonne Paris Cité, Paris, France; 9 Institut Jérôme Lejeune, Paris, France; 10 Lee Kong Chian School of Medicine, Nanyang Technological University, Singapore, Singapore

**Keywords:** Down syndrome, Alzheimer’s disease, Early endosomes, Endocytosis, Electron microscopy, Super-resolution microscopy

## Abstract

Enlarged early endosomes have been visualized in Alzheimer’s disease (AD) and Down syndrome (DS) using conventional confocal microscopy at a resolution corresponding to endosomal size (hundreds of nm). In order to overtake the diffraction limit, we used super-resolution structured illumination microscopy (SR-SIM) and transmission electron microscopies (TEM) to analyze the early endosomal compartment in DS.

By immunofluorescence and confocal microscopy, we confirmed that the volume of Early Endosome Antigen 1 (EEA1)-positive puncta was 13–19% larger in fibroblasts and iPSC-derived neurons from individuals with DS, and in basal forebrain cholinergic neurons (BFCN) of the Ts65Dn mice modelling DS. However, EEA1-positive structures imaged by TEM or SR-SIM after chemical fixation had a normal size but appeared clustered. In order to disentangle these discrepancies, we imaged optimally preserved High Pressure Freezing (HPF)-vitrified DS fibroblasts by TEM and found that early endosomes were 75% denser but remained normal-sized.

RNA sequencing of DS and euploid fibroblasts revealed a subgroup of differentially-expressed genes related to cargo sorting at multivesicular bodies (MVBs). We thus studied the dynamics of endocytosis, recycling and MVB-dependent degradation in DS fibroblasts. We found no change in endocytosis, increased recycling and delayed degradation, suggesting a “traffic jam” in the endosomal compartment.

Finally, we show that the phosphoinositide PI (3) P, involved in early endosome fusion, is decreased in DS fibroblasts, unveiling a new mechanism for endosomal dysfunctions in DS and a target for pharmacotherapy.

## Introduction

Early morphological alterations of subcellular organelles from the endosomal pathway have been extensively described in Alzheimer’s disease (AD) and Down syndrome (DS), notably an abnormal increase in the size of early endosomes in pyramidal neurons of the cortex [10]. Such alteration of the morphology of endosomes is still considered as the earliest neuropathological hallmark of AD since it occurs in the neocortex of patients with sporadic AD, in Familial early onset AD (FAD) with mutations in the gene encoding the Amyloid Precursor Protein (APP), and in DS individuals carrying a trisomy for human chromosome 21 (HSA21) before amyloid peptides deposition [9, 14]. APP gene maps to HSA21 and is triplicated in DS resulting in an overexpression of APP which is believed to increase the occurrence of AD in this population [7, 18]. Indeed individuals with DS develop typical symptoms of AD type dementia in their fifth decade of life, such as behavioral changes and memory deficits, early cerebral amyloid deposition confirmed by positron emission tomography (PET) using Pittsburgh Compound B (PiB) and Florbetapir, and change of Aβ peptides levels in plasma and cerebrospinal fluid [18, 36, 52, 62, 67, 84]. Neurons of individuals with DS harbor enlarged endosomes decades before the formation of amyloid deposits and the onset of AD clinical symptoms [14]. We and others have found enlarged endosomes in peripheral blood mononuclear cells (PBMC), lymphoblastoid cell lines (LCLs) and primary fibroblasts from individuals with DS [11, 20, 45] and AD [19]. In addition, previous studies showed endosomal enlargement in the brain of mouse models of FAD with mutations in the APP gene [16] and in Ts65Dn mice [12], the best-characterized and most widely used mouse model of DS.

APP-βCTF is suspected to be responsible for early endosome abnormalities in AD and DS. APP-βCTF favors APPL1 recruitment to early endosomes, which in turn stabilizes the active form of the Rab GTPase Rab5 leading to increased early endosome fusion [47]. Rab5 is a major regulator of endosome biogenesis and Rab5 overexpression increases intracellular amyloid β (Aβ) production [34]. Apart from APP, our team showed that the overexpression of SYNJ1 in DS is also implicated in early endosome enlargement [20]. Additionally, increased cholesterol in the brain of AD patients also triggers this phenotype [54]. While the role of APP is crucial in the development of endosomal abnormalities in AD, it should also be qualified in light of studies showing that APP alone might not be sufficient to explain all phenotypes related to endosomal dysfunction in AD and DS [20, 85]. Many other systems regulating the endo-lysosomal pathway are deregulated in AD and DS such as levels of phosphoinositides [8, 53, 58] and the retromer function [73], through the involvement of various risk factors and overexpressed genes respectively [8].

Measurements of early endosomal morphological characteristics in AD and DS relied on images obtained by conventional light microscopy with a resolution that is fundamentally limited by the diffraction of light at 200 nm at best, i.e. in the diameter range of early endosomes [26]. In order to fully characterize the endosomal compartment in DS, we used Electron Microscopy (EM) and super-resolution Structured Illumination Microscopy (SIM) operating beyond the diffraction-limited resolution [71]. We were able to revisit early endosome morphological alterations in peripheral cell models of DS (LCLs, fibroblasts), in the brain of Ts65Dn mice and in human neurons derived from isogenic induced pluripotent stem cells (iPSC) from an individual with mosaic trisomy 21. In all biological materials from DS condition analyzed by conventional confocal microscopy we confirmed a significant increase in the mean size of puncta labelled with a monoclonal antibody against Early Endosome Antigen 1 (EEA1) as compared to biological material from control condition. However, using either EM or Super-Resolution Structured Illumination Microscopy (SR-SIM), we showed that endosomes in the DS condition were not significantly bigger. Since all these techniques relied on the use of a fixative that can alter the morphology of organelles, we chose an additional method that preserves molecular and structural integrity of samples using vitrification by high pressure freezing (HPF). EM after HPF on unfixed fibroblasts from individuals with DS revealed that early endosomes are not significantly bigger but much more numerous as compared to fibroblasts from euploid controls. Interestingly RNAseq analyses from fibroblasts of individuals with DS and controls revealed expression changes of genes involved in cargo sorting from early endosomes to multivesicular bodies (MVB) toward the degradation pathway. We thus analyzed the dynamics of endocytosis, recycling and degradation in fibroblasts from individuals with DS and euploid controls. We show that MVB-dependent cargo sorting is impaired in DS, along with decreased PI (3) P levels in fibroblasts from individuals with DS, thus suggesting a new mechanism underlying pathological endosomal changes in DS.

## Materials and methods

### Cell culture

#### Lymphoblastoid cell lines (LCLs)

LCLs from euploid individuals and individuals with DS were provided by Institut Lejeune (Paris). LCLs were obtained by immortalization of lymphocyte B with Epstein-Barr virus according to Tlili et al. (2012) [77]. LCLs were cultured in OptiMEM (Gibco, Thermofisher Scientific) supplemented with 10% fetal bovine serum and 1% penicillin/streptomycin. All LCLs were karyotyped. They first underwent hypotonic shock with pre-heated potassium chloride at 5.6 g/L for 20 min at 37 °C, before two fixation steps in Carnoy (25% acetic acid, 75% methanol) of 20 min each. Fluorescence in situ hybridization (FISH) was then carried out using the centromeric probe of chromosome 21 (Cytocell) and trisomy 21 was searched on 50 cells per cell line.

#### Fibroblasts

Euploid line ID GM05659 and DS line ID AG05397 were purchased from Coriell Cell Repositories, DS lines ID TOM, FRA, GUC, SAA and BAQ were provided by the Institut Jérôme Lejeune and euploid lines ID 94 and 69 were provided by the ImaBio3 cohort (Supplementary Table). Cells were cultured as described previously [20].

Apolipoprotein E (APOE) genotype was determined for 2 N and DS fibroblasts used in Figs. 2, 3, 6, 7, 8 and 9 by PCR-based Sanger sequencing. Genomic DNA was extracted using Nucleospin Tissue kits from Macherey Nagel according to the manufacturer’s instructions. Exon 4 from APOE gene containing the SNP corresponding to the ε3/ε4 alleles was amplified using PCR with the following primers: APOE sense, 5′-TAAGCTTGGCACGGCTGTCCAAGGA-3′; APOE antisense, 5′-ACAGAATTCGCCCCGGCCTGGTACAC-3′. For each sample, the reaction mixture (50 μl) contained 200 ng of genomic DNA, 10 μl PCR Flexi buffer (5x), 3 μl MgCl_2_ (25 mM), 1 μl dNTPs (10 mM), 1 μl of each forward and reverse primers (10 μM), and 0.25 μl GO Taq DNA polymerase (Promega). The cycling program was carried out after a preheating step at 95 °C for 2 min and 35 cycles of denaturation at 95 °C for 1 min, annealing at 68 °C for 1 min and extension at 72 °C for 1 min. The amplified fragments were then purified and sequenced with the same primers. We obtained the following APOE genotypes: GM05659 (ε2 / ε3), 94 (ε3 / ε3), 69 (ε3 / ε3), AG05397 (ε3 / ε3), TOM (ε3 / ε3) and FRA (ε2 / ε4).

#### Human isogenic induced pluripotent stem cells (iPSCs)-derived neurons

Isogenic human T21 iPS cell lines were generated from consented surplus diagnostic samples of human skin fibroblasts using Sendai virus reprogramming and fully characterized and cultured as described previously in Murray et al. [60]. Neuronal differentiation was performed by first deriving neural stem cells (NSCs) using Life Technologies Neural Induction medium as per manufacturer’s protocol or the dual SMAD inhibition method as described in [60]. For terminal neuronal differentiation, NSCs at passages 7–9 were seeded onto poly-L-ornithine and laminin (Sigma Aldrich) coated glass coverslips (thickness of 0.17 ± 0.005 mm) for optical microscopies or plastic coverslips for electron microscopy at a density of 20,000–50,000 cells/cm^2^. The next day, the medium was switched to 3 N medium supplemented with 10 ng/ml of BDNF and GDNF (Peprotech), 1 mM cAMP and 200 nM ascorbic acid (Sigma Aldrich). Fresh medium was changed twice a week and cells were fixed for analysis at days 60–65 post terminal differentiation. iPSC-derived neurons are glutamatergic excitatory neurons as described in Murray et al. [60]. We analyzed cells from the euploid clone C3 and the DS clone C5.

### Animals

F1 Ts65Dn mice were obtained from the Jackson Laboratory and maintained on a B6C3HF1 background by breeding Ts65Dn/B6EiC3 females (SN005252) with B6EiC3 males (SN003647). Only male Ts65Dn mice were used in the study, as differences in basal forebrain cholinergic neurons (BFCNs) size and number were observed between Ts65Dn males and females [46]. We used 4-month-old mice: 4 wild-type (WT) mice and 5 Ts65Dn mice.

### Immunofluorescence

#### Immunocytochemistry on iPSCs-derived neurons and fibroblasts

Cells were fixed in paraformaldehyde 4% for 20 min. After paraformaldehyde quenching (NH_4_Cl 50 mM), permeabilization (Triton X-100 0.2% in PBS) and blocking (bovine serum albumin 3% in PBS) steps, cells were incubated 1 h in primary antibodies (rabbit anti-EEA1 primary antibody C45B10, Cell Signaling, 1/500; mouse anti-MAP 2 antibody, MAB3418, Millipore, 1/500; mouse anti-Phosphatidylinositol-3-phosphate (PI (3) P) antibody, Z-P003, Echelon Biosciences, 1/200). Cells were rinsed and incubated in anti-rabbit AlexaFluor-488 and anti-mouse AlexaFluor-568 secondary antibodies for 1 h (1/1000, Invitrogen), counterstained with DAPI (1 μg/mL, Vector Laboratories), rinsed and mounted in Fluoromount-G for confocal microscopy or Vectashield (Vector Laboratories) for SIM. For confocal microscopy, z-stacks images (1024 × 1024 pixels, representing voxels of 0.0451 × 0.0451 × 0.198 μm) were taken using a Leica TCS SP8 AOBS confocal microscope with a 63x/NA = 1.40 oil immersion objective and × 4 zoom at ICMQuant facility. For fibroblasts, we analyzed between 26 and 32 cells in each individual, in a total of 3 euploid individuals and 3 individuals with DS.

#### Immunohistochemistry

4-month-old WT (*n* = 4) and Ts65Dn (*n* = 5) mice were anaesthetized by intraperitoneal injection of pentobarbital (60 mg/kg) and transaortically perfused with paraformaldehyde 4% / glutaraldehyde 0.05% / phosphate buffer 0.1 M. Brains were dissected and post-fixed in paraformaldehyde 4% / glutaraldehyde 0.05% / phosphate buffer 0.1 M overnight at 4 °C and then transferred in PBS at 4 °C until sectioning. 40 μm-thick coronal sections were obtained using a vibratome (Microm) and stored in PBS / sodium azide 0.4% until staining.

Free floating brain sections were washed three times in PBS and pre-incubated in blocking and permeabilization solution (normal donkey serum 5% / Triton X-100 0.2% in PBS) for 1 h. Brain sections were incubated overnight in primary antibody solution: goat anti-choline-acetyltransferase (ChAT) antibody (polyclonal, AB144P, Millipore, 1/300, kindly provided by Dr. Sylvie Berrard, Hôpital universitaire Robert-Debré, Paris, France), mouse anti-NeuN antibody (monoclonal, MAB377, Millipore, 1/500) and rabbit anti-EEA1 antibody (C45B10, Cell Signaling, 1/400). Tissues were rinsed in normal donkey serum 5% / Triton X 100 0.2% / PBS and then incubated with secondary fluorescent polyclonal antibodies: AlexaFluor 488 donkey anti-rabbit, AlexaFluor-555 donkey anti-goat and AlexaFluor-647 donkey anti-mouse (Molecular Probes, 1/500). Brain sections were rinsed in phosphate buffer 0.1 M, stained with DAPI (10 μg/mL) and mounted on Superfrost Plus slides (Thermo Scientific) in Fluoromount G. Samples were imaged by confocal microscopy, using a Leica TCS SP2 AOBS and Leica TCS SP8 with a 63x, NA = 1.40 oil immersion objective and × 3 zoom (single z-scans, 1024 × 1024 pixels, representing pixels of 0.077 × 0.077 μm) at PICPS. Images were taken at the cell equatorial section, where the nuclear surface is the largest. Three brain sections from each mouse were used and 24 neurons were analyzed per neuronal type and per mouse (8 neurons from each brain section). The brain sections were selected to provide a representative sampling of the entire population of BFCNs. Thus, we selected one anterior section, one medial section and one posterior section in each mice.

### Electron microscopy

#### Pre-embedding immunocytochemistry

Early endosomes of LCLs and fibroblasts were labelled by anti-EEA1 antibody (C45B10, Cell Signaling) with pre-embedding immunoperoxidase cytochemistry and observed as described previously [54].

#### Pre-embedding immunohistochemistry of basal forebrain cholinergic neurons

4-month-old WT and Ts65Dn mice were anaesthetized (pentobarbital 60 mg/kg) and transcardially perfused with paraformaldehyde 4% / glutaraldehyde 0.1% / phosphate buffer 0.1 M. Dissected brains were further post-fixed in paraformaldehyde 4% / sucrose 15% in PBS for 2 h at 4 °C. 70 μm-thick coronal sections were obtained using a vibratome (Microm). After glycine aldehyde 0.1 M quenching and H_2_O_2_ 0.3% blocking of endogenous peroxidase, sections were treated with bovine serum albumin 5% / normal donkey serum 5% in PBS, and further incubated in anti-EEA1 (C45B10, Cell Signaling, 1/500) and anti-ChAT (AB144P, Millipore, 1/200) in PBS overnight at room temperature. For ChAT labelling, incubation in donkey anti-goat conjugated to ultra-small gold (Aurion, Netherlands, 1/50) was followed by extensive washings, 10 min post-fixation in glutaraldehyde 2% and finally a silver enhancement reaction (HQ Silver, NanoProbes). For EEA1 labelling a biotinylated anti-rabbit IgG (Vector, CA, USA) was applied as secondary antibody, followed by ABC peroxidase complex amplification (Vectastain Elite, Vector, CA, USA) and revelation was performed with diaminobenzidin (DAB) 0.05% as the chromogen. After OsO_4_ 1% post-fixation, dehydration in graded acetone including a uranyl 1% staining step in acetone 70% preceded embedding in Epon resin. Ultrathin sections were lightly stained with lead citrate, observed using a Philips CM120 electron microscope (Philips, Eindhoven, The Netherlands) operated at 80 kV, images were recorded with a Morada digital camera (Olympus Soft Imaging Solutions GmbH, Münster, Germany), and measures were taken with the associated iTEM software.

#### Electron microscopy after high-pressure freezing (HPF)

Cells were cultured on CryoCapsules (CryoCapCell, France) following the protocol described previously [37, 38], for 3 days before vitrification. Cells were vitrified by High Pressure Freezing on an HPM Live μ (CryoCapCell, France) followed by freeze-substitution. Using an Automated Freeze Substitution machine (AFS-2, Leica microsystem, Austria), samples were dehydrated at − 90 °C in dry acetone / uranyl acetate 0.05% / distilled water 5% / glutaraldehyde 0.01% for 2 h. The temperature was raised to − 45 °C at 5 °C / hour and rinses in dry acetone 3 times before progressive impregnation in Lowicryl HM20 (EMS diasum, 25, 50, 75, 100%, in acetone, in steps of 2 h and the last step of 100% HM20 overnight) before initiating polymerization at − 45 °C for 48 h. The temperature was finally raised to + 20 °C under UV before collection at room temperature for further ultrathin sectioning and TEM observation.

### 3D structured illumination microscopy (SIM)

Super-resolution light microscopy was performed on a Zeiss ELYRA SIM microscope, equipped with a Plan-Apochromat 63×/1.40 NA oil-immersion objective (Carl Zeiss). The illumination patterns of the 405, 488 and 561 nm lasers were projected into the sample. The emitted fluorescence light was detected with an EMCCD camera (iXon 885, Andor Technology). Five phase translations and three rotations of the illumination pattern were recorded at each z-plan and image stacks (120-nm increment along z axis) were acquired. The 3D stacks were then computationally reconstructed with the ZEN imaging software package (algorithm of Heintzmann and Cremer, [39]) to generate super-resolution 3D SIM (SR-SIM) images with twofold extended resolution in the three axes (reconstructed image format = 1904 × 1900 pixels, representing voxels of 0.04 × 0.04 × 0.12 μm).

### Image quantification

Images were analyzed and quantified using the Spot Detector plugin of Icy (biological images analysis suite; http://icy.bioimageanalysis.org) [25]. This plugin identifies subcellular organelles such as endosomes and measures their areas in 2D images or their volumes in 3D images using an undecimated wavelet transform [19]. ROI was delineated around the cells, on the z-plan where they appeared as the largest. For human neurons derived from iPSC, we manually counted early endosomal clusters in SR-SIM images based on binary images resulting from the Spot Detector plugin of Icy. We considered that a cluster is constituted of at least 3 early endosomes.

For EM following HPF, we counted endosomes area and number / μm^2^ of cytoplasm (numerical density) on × 9700 magnification images in Icy. The area of cytoplasm excludes the area of the nucleus. Between 15 and 27 cells were counted in each of 2 euploid (GM05659 and 94) and 3 DS fibroblasts lines (AG05397, TOM and FRA). Without a specific staining, early endosomes identification relied on morphological characteristics described in the literature [79] and on the aspect of EEA1-positive early endosomes observed by EM after aldehyde fixation in fibroblasts (Fig. 2d-g). We counted the objects presenting a vacuolar region transparent to electrons, devoided of a clathrin-type coat surrounding the whole vesicle, sometimes presenting tubular extensions, containing a maximum of 3 intraluminal vesicles or filamentary material.

In fibroblasts, we measured the mean fluorescence intensity per cell of the transferrin receptor and PI (3) P stainings. PI (3) P was immunostained with an antibody previously used to measure PI (3) P signal intensity level [28]. We performed z-projections of each image to obtain a sum of pixel intensities in a single plan and substracted the total pixel intensity per cell to the mean background intensity measured from 3 different ROI in each image.

### RNASeq analysis

Total RNA was extracted from fibroblasts from 3 euploid individuals and 6 individuals with DS using NucleoSpin RNA II kit from Macherey Nagel according to the manufacturer’s instructions.

Messenger (polyA+) RNAs were purified from 500 ng of total RNA using oligo (dT). Libraries were prepared using the strand-specific RNA-Seq library preparation KAPA mRNA Hyperprep (Roche). Libraries were multiplexed by 5 on 2 mid output flowcells. A 75-bp paired-end read sequencing was performed on a Nextseq500 sequencer (Illumina). The mean number of reads passing the Illumina quality filter was 33 ± 6 million per sample.

Sequenced reads passing the quality controls using FastQC and Trimmomatic were aligned to the human reference genome hg19using Galaxy (https://usegalaxy.org/). For the RNA-Seq dataset, all genes with fewer than 10 raw read counts across 75% of the samples (low expressed genes) were filtered out from the analysis. Expression data was then normalized using the rlog (regularized logarithm) function in the R package DESeq2 [50] and differential analysis was performed with a FDR correction. Only genes that had a corrected *p*-value < 0.05 were considered to be differentially expressed. Gene ontology (GO) enrichment analysis - including KEGG pathways, biological processes, and chromosomal location – were performed on the differentially expressed genes using the EnrichR web tool [15]. All RNASeq analysis and graphs were performed with R 3.6.0, with the pheatmap package used to generate the heatmap. Among 3469 GO (Gene Ontology) categories (2018) from EnrichR, we selected the categories containing the terms *endocytosis* (corresponding to 12 GO entries) and the term *recycling* (corresponding to 5 GO entries). In KEGG (2019), one entry corresponded to the term *endocytosis* and no entry corresponded to the term *recycling*. From a total of 1084 differentially expressed gene, 44 differentially expressed genes are identified as involved in endocytosis and recycling based on GO categories.

### Epidermal growth factor receptor (EGF-R) degradation

Fibroblasts were serum-starved for 6 h before incubation with human EGF (E9644, Sigma) at 37 °C for 30, 60, 90 and 120 min (50 ng/mL in DMEM/GlutaMax /1% penicillin / streptomycin). Times 0 corresponds to non-treated cells collected after serum depletion. EGF-R levels in cell lysates were quantified by western blot. We performed EGF treatment on 3 euploid and 3 DS fibroblast lines, once for each line. The experiments were split in three series of one euploid and one DS line (Experiment 1: GM05659 vs. AG05397 / Experiment 2: 94 vs. TOM / Experiment 3: 69 vs. FRA). Each experiment was analyzed by western blot.

### Western blots

Fibroblasts lysates were prepared in RIPA buffer (R0278 Sigma) with complete protease inhibitor cocktail (Roche). Protein concentration in each sample was determined with Bradford protein assay and equal quantity of protein was loaded in Mini-PROTEAN 4–20% Tris-Glycine SDS-PAGE (Biorad) and transferred on nitrocellulose membranes (Santa Cruz). After incubation in TBS/non-fat milk 5% for 1 h, membranes were incubated in anti-EGF-R (ab52894, Abcam, 1/1000) or anti-GAPDH (CB1001, Millipore, 1/6000) diluted in TBS/Tween 0.1%/BSA 5% overnight at 4 °C. Membranes incubated in horseradish peroxidase conjugated secondary antibodies anti-rabbit (1:5000, 31,460, Thermofisher Scientific) or anti-mouse (1:5000, 31,430, Thermofisher Scientific) diluted in TBS/Tween 0.1% for 1 h, incubated in ECL substrate (34,580, Thermofisher Scientific) and developed on ECL films (Amersham Hyperfilm, GE). EGF-R level was normalized to GAPDH level in each condition.

### Flow cytometry

Transferrin internalization protocol was adapted from literature [49, 65, 76]. Fibroblasts were plated in 25cm^2^ flasks 48 h before the experiment. Fibroblasts were kept at 4 °C for 15 min before incubation in human transferrin-Alexa Fluor 647 (25 μg/mL, T23366, Thermofisher Scientific) in serum-free DMEM/GlutaMax (Gibco, Thermofisher Scientific) at 37 °C for 4 min. Cells were trypsinized and resuspended in DMEM GlutaMax supplemented with fetal bovine serum 10% and penicillin-streptomycin 1%. All the following steps are performed at 4 °C or on ice. Cells were centrifuged 5 min at 100 g and were resuspended in PBS containing cell death marker (NucGreen Dead 488, R37109, Invitrogen). NucGreen is a cell-impermeant nucleic acid stain that emits fluorescence when bound to DNA. The NucGreen marker can only enter the cells that have lost plasma membrane integrity. Cells were then centrifuged 5 min at 100 g, resuspended in 1 mL of paraformaldehyde 1% and incubated for 10 min. Ten milliliters of PBS were directly added to paraformaldehyde, cells were centrifuged 5 min at 100 g and resuspended in 250 μL of PBS to obtain a concentration of 1.10^6^ cells/mL. Cells were then analyzed at the flow cytometer MACSQuant (Miltenyi Biotec) of the Cyto-ICAN facility. The data was analyzed with Flowlogic (Miltenyi Biotec). The gating strategy excludes doublets and cells positive for the death marker.

### Transferrin receptor recycling

The transferrin receptor recycling protocol was adapted from the literature [49, 76]. Fibroblasts were plated on glass coverslips 48 h before the experiment. After rinsing with PBS, fibroblasts were kept at 4 °C for 30 min in serum-free DMEM/GlutaMax (Gibco, Thermofisher Scientific). Fibroblasts were then rinsed on ice and incubated in DMEM / GlutaMax / SVF 10% / penicillin-streptomycin 1% supplemented with transferrin (100 μg / mL, T13342, Thermofisher Scientific) at 37 °C for 15 min. Cells were rinsed on ice and fixed in PFA 4% for 10 min. Cells were then immunolabelled against the surface transferrin receptor, therefore with an immunolabelling protocol without permeabilization. Apart from permeabilization, the staining protocol is identical to that presented above. Cells incubated 1 h in primary antibody anti-CD71 (monoclonal rabbit, 13,208, Cell Signaling, 1/300) and 1 h in secondary antibody anti-mouse Alexa Fluor 647 (donkey, A^− 31,571^, ThermoFisher Scientific, 1/1000) were then observed under a Leica TCS SP8 AOBS confocal microscope with an oil immersion objective 63x / NA = 1.40 on the ICMQuant platform.

### Statistical analysis

Data extracted from Icy were analyzed with Statistica and GraphPad Prism.

#### Comparison of the mean area or volume of early endosomes

We tested the genotype effect on the size of early endosomes imaged by EM and confocal microscopy in LCLs, fibroblasts and human neurons derived from iPSCs using a mixed effects nested analysis of variance (ANOVA), the random cell factor being nested in the random individual factor, itself nested in the fixed genotype factor. Thus, we could test a global difference of early endosome size between genotypes despite the important variability of mean early endosome size and number between cells and individuals. When required for the normality and homoscedasticity assumptions to be true, size measures were analyzed after logarithmic transformation.

#### Comparison of the mean number of early endosomes

In fibroblasts and neurons derived from iPSC, the volume of the cell or soma varies importantly between cells and is hardly measurable. To consider the size of each cell in the analysis, we divided the number of early endosomes per cell by the ROI area. In confocal and electron microscopy, the number of early endosomes was normalized to the ROI area (number/μm^2^) and was analyzed with mixed effect ANOVA, the random individual factor being nested in the fixed genotype factor. In EM after HPF, the number of early endosomes were normalized to the area of cytoplasm, excluding the nucleus area. For neurons derived from iPSCs, euploid and T21 cells coming from a single mosaic individual, there is no individual effect. Thus, the number of early endosomes were adjusted to the ROI area (number/μm^2^) measured by confocal microscopy and the number of clusters normalized to the number of early endosomes measured in SR-SIM were compared between genotypes using Mann-Whitney test.

#### Comparison of the mean levels of EGF-R by western blot

The levels of EGF-R obtained by western blot were compared between euploid (2 N) and DS fibroblasts with a two-way ANOVA (the two factors being genotype and time-point) followed by a *post-hoc* multiple comparison between genotypes for each time-point with Bonferroni correction for multiple testing.

#### Comparison of the mean fluorescence intensity of transferrin receptor in flow cytometry

The mean fluorescence intensity of internalized fluorescent transferrin measured by flow cytometry was compared between 2 N and DS fibroblasts with a Mann-Whitney test.

#### Comparison of the mean fluorescence intensity of recycled transferrin receptor in flow cytometry

The mean fluorescence intensity of recycled fluorescent transferrin measured by confocal microscopy was compared between 2 N and DS fibroblasts with a Mann-Whitney test.

#### Comparison of the mean fluorescence intensity of PI (3) P

The mean fluorescence intensity of PI (3) P measured by confocal microscopy was compared between 2 N and DS fibroblasts with a two-way ANOVA, the random individual factor being nested in the fixed genotype factor.

## Results

### Ultrastructure of EEA1-positive early endosomes in LCLs from individuals with DS

Using confocal microscopy, we have previously shown that LCLs from individuals with DS contain enlarged EEA1-and Rab5-positive puncta as compared to euploid individuals [20]. The limit of resolution of conventional light microscopy being in the diameter range of early endosomes, we wished to analyze the ultrastructure of early endosomes using EM. We imaged 50–70 nm-thick sections following pre-embedding immunocytochemistry with anti-EEA1 antibody and DAB staining. EEA1 electron-dense DAB precipitates were found surrounding the cytoplasmic leaflet of early endosome membranes and extending at a few nanometers from endosome profiles, as previously described [83].

**Fig. 1:**
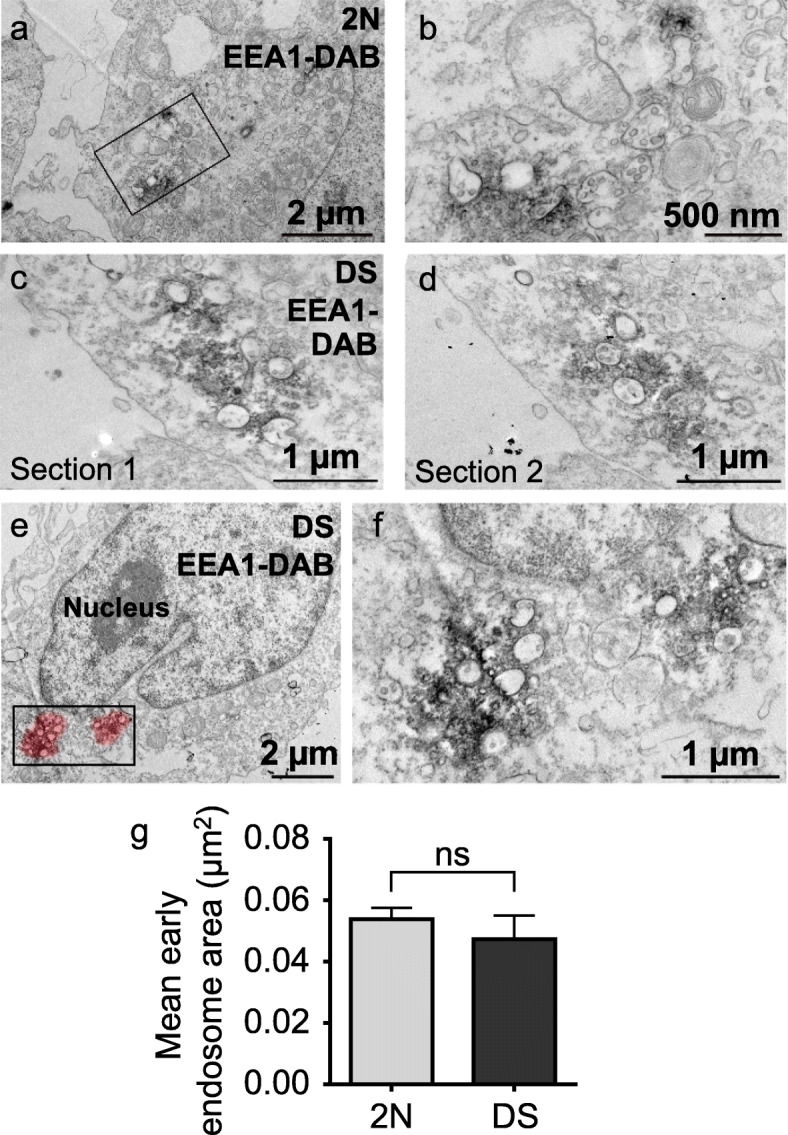
Ultrastructural imaging of early endosomes in LCLs from individual with DS. **a**, **b** Electron micrograph representing EEA1 immunoperoxidase-labelled early endosomes in a LCL from a euploid individual. The squared zone in (**a**) is magnified in (**b**); **c**, **d** 2 serial TEM sections of an EEA1 immunoperoxidase-labelled cluster of early endosomes in a trisomic lymphoblastoid cell; **e**, **f** 2 clusters of early endosomes in a lymphoblastoid cell from an individual with DS. The pseudo-colored red halo suggests the extent of labelled area as it should appear with confocal analysis. The squared zone in (**c**) is enlarged in (**d**); **g** Quantification of early endosomal area in LCLs imaged by TEM, showing no significant difference in mean early endosomal area between LCLs from euploid individual and individual with DS (mixed effects ANOVA, genotype, *p*-value = 0.57)

EEA1-positive early endosomes were found clustered in LCL from individuals with DS (Fig. 1c-f). Clusters of early endosomes were occasionally observed in LCLs from euploid individuals but at lower incidence and with less endosomes involved than in LCLs from individuals with DS (Fig. 1a, b). Observation of serial sections enabled to identify clusters of early endosome profiles that were fully disconnected from one another and distinct from smaller surrounding tubule or vesicular profiles (Fig. 1c, d; suppl. Fig. 1). These clusters of early endosomes had a diameter of approximately 1 μm, as illustrated by the pseudo-colored halos (Fig. 1e). Interestingly, this diameter range corresponded to measures of enlarged endosomes previously described with confocal microscopy [20]. Morphometric analysis revealed that mean early endosomal area was not significantly different between LCLs from euploid individuals (*n* = 28 cells, mean area = 0.054 μm^2^, SEM ± 0.0037) and individuals with DS (*n* = 43 cells, mean area = 0.047 μm^2^, SEM ± 0.0078) (mixed effects ANOVA, genotype *p*-value = 0.57) (Fig. 1g).

### Morphology of early endosomes in fibroblasts from individuals with DS studied by confocal and electron microscopy

**Fig. 2:**
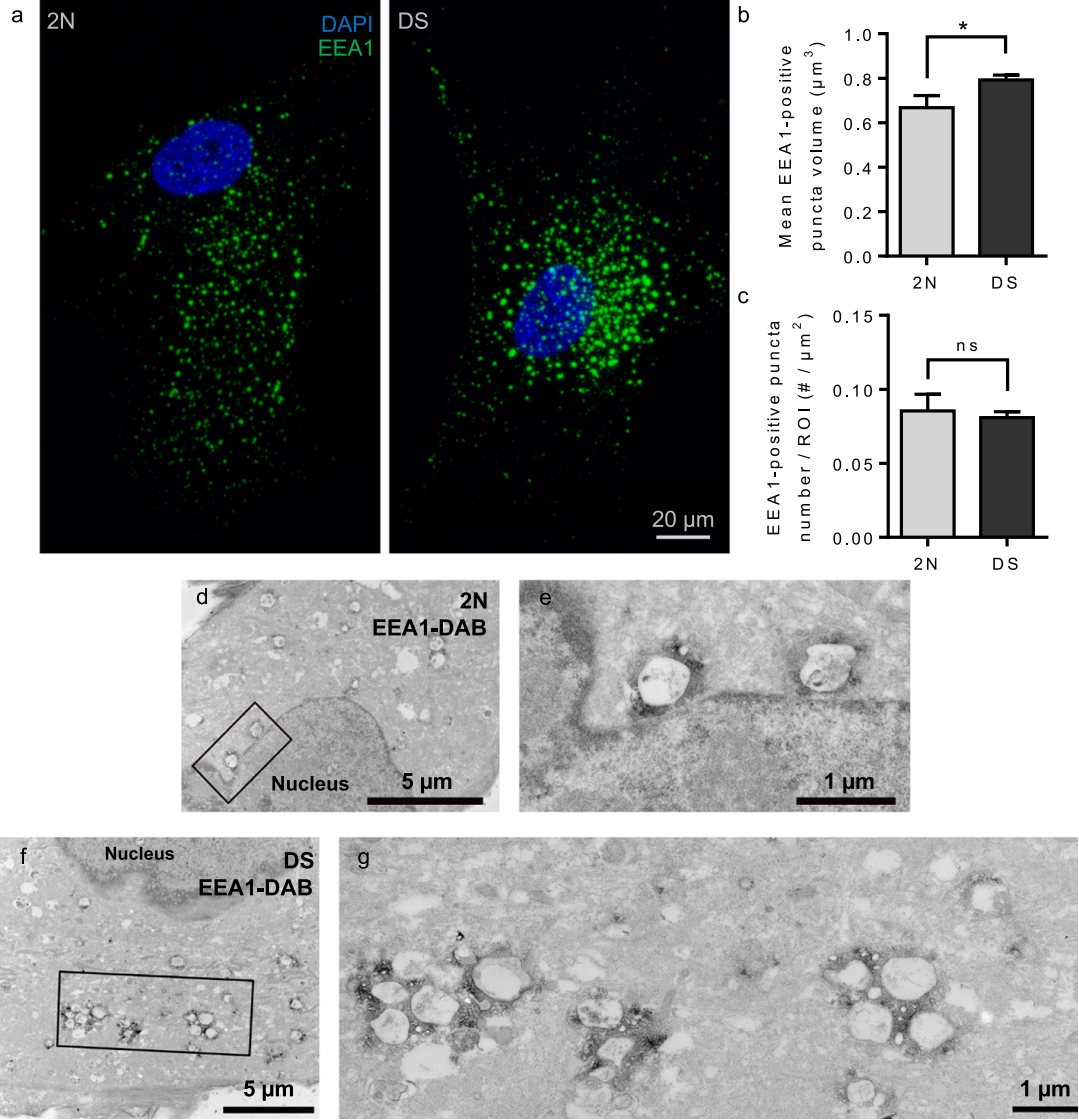
Confocal microscopy and ultrastructural imaging of early endosomes in fibroblasts from individuals with DS. **a** Representative images of EEA1-positive early endosomes (green) in fibroblasts from euploid individuals and individuals with DS; **b** Quantification of EEA1-positive puncta volume from confocal microscopy images of fibroblasts, showing a significantly increased volume of early endosome in DS condition (mixed effect ANOVA, genotype *p-value = 0.03); **c** EEA1-positive puncta number normalized to ROI area is not significantly different between euploid and DS fibroblasts in confocal microscopy (mixed effect ANOVA, ns, genotype *p*-value = 0.73); **d**-**g** Electron micrographs of EEA1 immunoperoxidase-labelled fibroblasts from a euploid individual (**d** squared zone magnified in (**e**)) and an individual with DS (**f** squared zone magnified in (**g**)) after chemical fixation

Using confocal microscopy, we analyzed the size and number of EEA1-positive puncta in fibroblasts from three individuals with DS and three controls from various cell banks (Supplementary Table). By confocal microscopy, we found that EEA1-positive puncta volume was significantly increased in fibroblasts from individuals with DS (*n* = 3 individuals, mean volume = 0.79 μm^3^, SEM ± 0.022) when compared to euploid fibroblasts (*n* = 3 individuals, mean volume = 0.67 μm^3^, SEM ± 0.0544) (mixed effects ANOVA, genotype *p*-value = 0.03) (Fig. 2a, b). The number of EEA1-positive puncta was not significantly different between fibroblasts from individuals with DS (*n* = 3 individuals, number/ROI area = 0.081 EEA1-positive puncta/μm^2^, SEM ± 0.0041) and euploid fibroblasts (*n* = 3 individuals, mean number/ROI = 0.085 EEA1-positive puncta/μm^2^, SEM ± 0.011) (mixed effects ANOVA, genotype *p*-value = 0.73) (Fig. 2c). This result was in agreement with previous studies published [11, 20, 45].

**Fig. 3:**
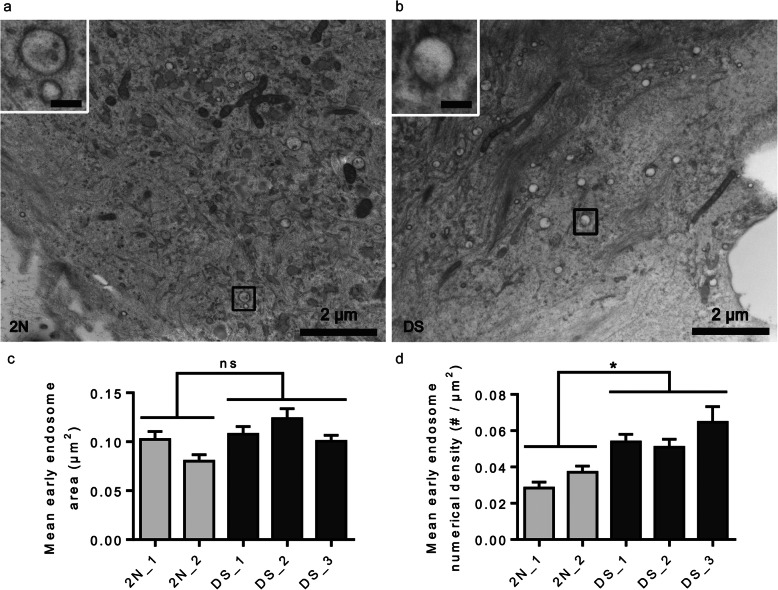
Electron microscopy after high-pressure freezing in fibroblasts from individuals with DS. **a** Electron micrograph of a euploid fibroblast (2N_2) and a fibroblast from an individual with DS (DS_1). Squared zones are enlarged in top left corners (scale bar = 500 nm); **b** Quantification of early endosomal area shows no significant difference between euploid and DS fibroblasts (mixed effects ANOVA, genotype *p*-value = 0.49); **c** Quantification of early endosome numerical density reveals that early endosomes are significantly more dense in fibroblasts from individuals with DS (mixed effects ANOVA, genotype *p-value = 0.031)

We next explored early endosomal ultrastructure by EM after pre-embedding immunocytochemistry with anti-EEA1 antibody and DAB staining. Fibroblasts from individuals with DS showed clusters of early endosomes (Fig. 2f, g). As in LCL, smaller clusters of early endosomes were also occasionally observed in fibroblasts from euploid individuals (Fig. 2d, e). In these experiments, aldehyde fixation could alter the ultrastructure of early endosomes as previously suggested [59]. In order to avoid aldehyde fixation of the cells, we performed EM after High-Pressure Freezing (HPF) (Fig. 3). Vitrification of the cells by HPF has been shown to preserve the ultrastructure of cellular components, enabling observations of close-to-native structures [59]. We imaged between 15 and 27 fibroblasts from 2 euploid and 3 individuals with DS and measured the number and surface of early endosomes based on morphological criteria. Representative images of fibroblasts from individual with DS (Fig. 3b) suggested that the number of early endosomes was increased as compared to fibroblasts from 2 N individuals (Fig. 3a), while their size appeared unchanged. In addition, the clustering of early endosomes appeared much less prominent as compared to EM pictures following aldehyde fixation (Fig. 3b as compared to Fig. 2f, g).

We measured the surface of early endosomes and did not find any significant difference between 2 N fibroblasts (*n* = 2 individuals, mean area = 0.091 μm^2^, SEM ± 0.011061) and fibroblasts from individuals with DS (*n* = 3, mean area = 0.11 μm^2^, SEM ± 0.0068) (mixed effects ANOVA, genotype *p*-value = 0.49) (Fig. 3c). However, we found a significant increase of the numerical density of early endosomes in fibroblasts from individuals with DS (*n* = 3 individuals, mean numerical density = 0.058 / μm^2^, SEM ± 0.004) as compared to 2 N (*n* = 2 individuals, mean numerical density = 0.033 / μm^2^, SEM ± 0.0044) (mixed effects ANOVA, genotype *p*-value = 0.031) (Fig. 3d).

### EEA1-positive early endosomes of BFCN of the Ts65Dn mouse model of DS

**Fig. 4:**
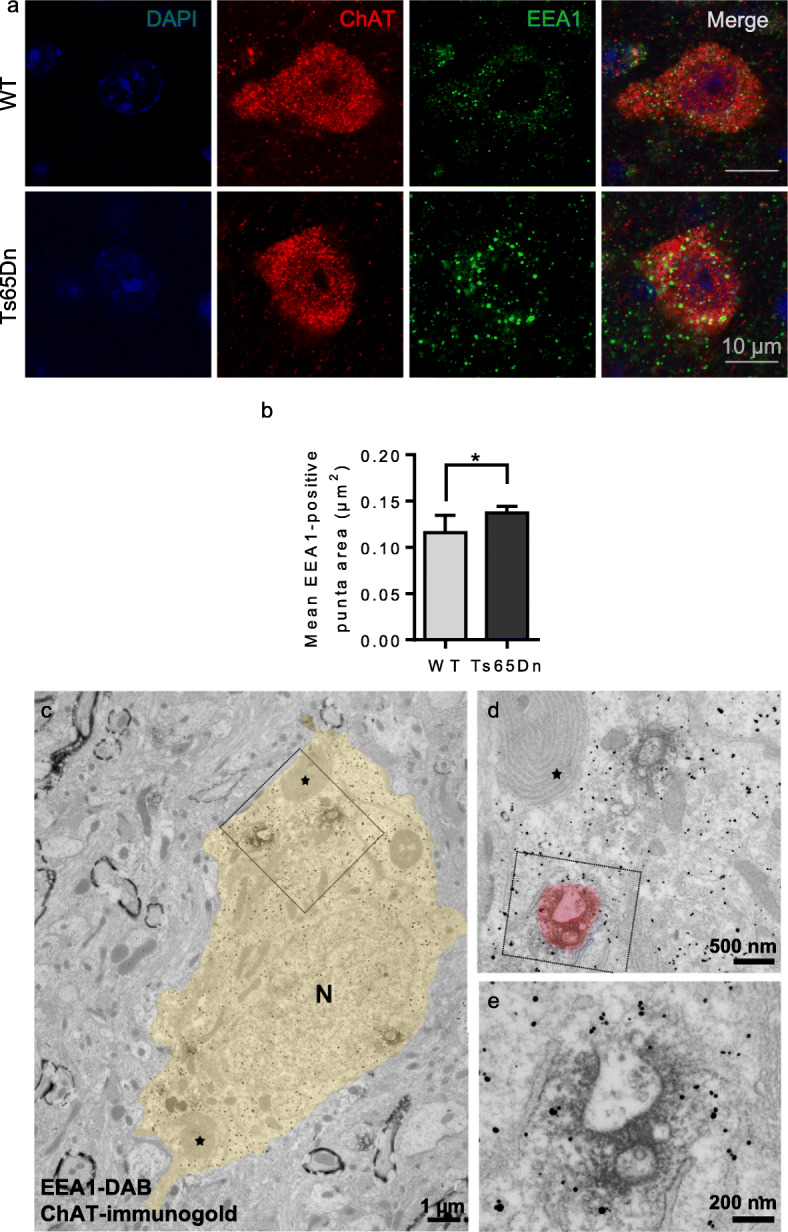
Confocal microscopy and TEM imaging of BFCNs from 4-month-old Ts65Dn mice. **a** Representative confocal images of DAPI (blue), ChAT (red), EEA1 (green) labelling and overlay in BFCNs from WT and Ts65Dn mice. For clarity, NeuN staining is not shown; **b** Quantification of EEA1-positive puncta surface at equatorial section of BFCNs shows a significant increase in mean area in Ts65Dn mice as compared to WT mice (mixed effects ANOVA, genotype *p-value = 0.04); **c** Low magnification micrograph of a ChAT gold-labelled BFCNs of a Ts65Dn mouse. Early endosomes are labelled by an EEA1-DAB precipitate. The soma profile is pseudo-colored in yellow for clarity. N indicates the nucleus and asterisks show two lamellar bodies, an organelle specifically found in cholinergic neurons. The squared zone is enlarged in (**d**) showing an isolated DAB-EEA1-labelled endosome and a cluster of at least 2 endosomes, the latter being magnified in (**e**). The pseudo-colored red halo in (**e**) suggests the extent of labelled area as it should appear with confocal analysis

Next we sought early endosome ultrastructural alterations in the brain of Ts65Dn mice, the most widely used mouse model of DS. We looked in basal forebrain cholinergic neurons (BFCNs) that show age-related degeneration in both AD and DS which may be caused by extracellular accumulation of Aβ or intracellular aggregation of hyperphosphorylated Tau and are responsible for cognitive impairment [4, 6, 32]. Ts65Dn also display age-related degeneration of BFCN at 5–6 months of age [40, 70]. We immunolabelled early endosomes using anti-EEA1 antibody on coronal sections of 4-month-old euploid and Ts65Dn mice, before age-related BFCNs loss (Fig. 4a). All measurements were performed in the soma, at the equatorial section. BFCNs were identified using an anti-choline-acetyltransferase (ChAT) antibody. We found that the mean EEA1-positive puncta area was significantly higher in BFCNs from Ts65Dn mice (*n* = 5 mice, mean area = 0.14 μm^2^, SEM ± 0.0075) as compared to BFCNs from euploid mice (*n* = 4 mice, mean area = 0.12 μm^2^, SEM ± 0.018) (mixed effects ANOVA, genotype *p*-value = 0.043) (Fig. 4a, b). These data obtained by confocal microscopy quantitatively confirm EEA1-positive puncta enlargement in BFCNs of 4-month-old Ts65Dn mice.

We then analyzed at the ultrastructural level EEA1-positive early endosome morphology in gold-labelled ChAT-positive BFCNs in 4-month-old euploid and Ts65Dn mice (Fig. 4c-e). Eleven cholinergic neurons in a Ts65Dn mouse were studied along serial sections through a 1 μm thickness where 20 endosome clusters containing two to three individual endosomes were found, with a mean ratio of 1.82 clusters per cholinergic neuron. As a comparison, we analyzed 20 cholinergic neurons in a euploid littermate along serial sections through a 1.32 μm thickness and found a total number of 14 clusters of early endosomes, with a mean ratio of 0.7 clusters per cholinergic neuron. Thus, we found a 2.6-fold increase of endosomal cluster occurrence in BFCNs of the Ts65Dn mouse as compared to BFCNs of the euploid littermate. Abnormal early endosome clustering is hence observed in BFCNs of the Ts65Dn mouse, indicating that early endosomes are prone to clustering in the brain of a DS mouse model, before BFCNs degeneration.

### EEA1-positive puncta in isogenic human neurons derived from induced pluripotent stem cells (iPSC) from an individual with mosaic trisomy 21 studied by confocal and SR-SIM

We questioned whether early endosome morphological abnormalities could be found in human neurons derived from induced pluripotent stem cells (iPSC) clones from an individual with a mosaic trisomy 21 [60], allowing direct comparison between isogenic non-trisomic (euploid) and trisomic (T21) conditions.

**Fig. 5:**
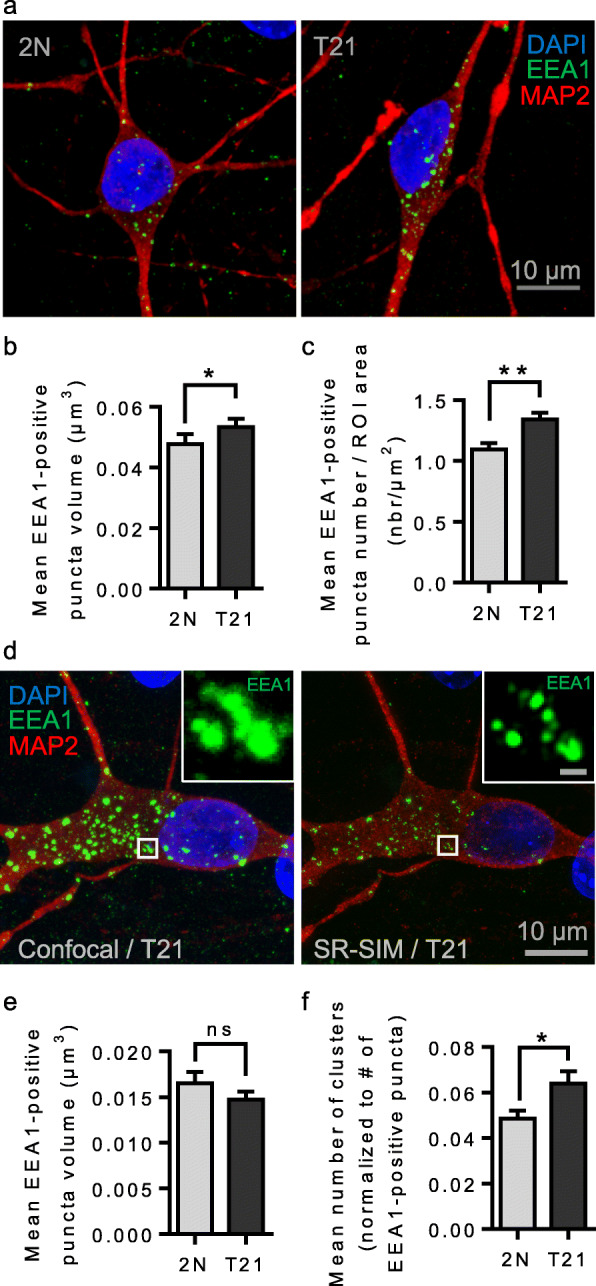
Confocal microscopy and SR-SIM of human isogenic iPSCs-derived neurons from an individual with T21 mosaicism. **a** Confocal z-projected representative images of DAPI (blue), MAP 2 (red), EEA1 (green) labelling in euploid and isogenic T21 iPSCs-derived neurons; **b** Quantification of the mean EEA1-positive puncta volume in confocal microscopy images reveals significantly increased volume in T21 neurons as compared to euploid isogenic neurons (mixed effects ANOVA, genotype *p-value = 0.03); **c** Quantification of EEA1-positive puncta number normalized to the ROI area shows a significant increase (Mann-Whitney test, **p-value = 0.0043); **d** Representative z-projected images of a T21 neuron imaged by confocal microscopy (left) and SR-SIM (right). A cluster of EEA1-positive puncta observed by SR-SIM appearing as enlarged puncta by confocal microscopy is magnified (scale bar = 500 nm); **e** Quantification of EEA1-positive puncta volume in SR-SIM images shows no significant difference (mixed effects ANOVA, genotype, *p*-value = 0.95); **f** The mean number of EEA1-positive puncta clusters per cell is significantly increased in T21 neurons as compared to euploid isogenic neurons (Mann-Whitney test, *p-value = 0.047)

By confocal microscopy, EEA1-positive puncta volume was significantly increased in T21 neurons (*n* = 20 cells, mean volume = 0.053 μm^3^, SEM ± 0.0028) as compared to euploid isogenic neurons (*n* = 20 cells, mean volume = 0.048 μm^3^, SEM ± 0.0033) (mixed effects ANOVA, genotype *p*-value = 0.03) (Fig. 5a, b). In addition, the number of EEA1-positive puncta adjusted to the ROI area (cell body area on the z-plan where it appeared as the largest) was significantly increased in T21 neurons (*n* = 20 cells, mean number/ROI area = 1.34 EEA1-positive puncta/μm^2^, SEM ± 0.055) as compared to euploid isogenic neurons (*n* = 20 cells, mean number/ROI area = 1.09 EEA1-positive puncta/μm^2^, SEM ± 0.054) (Mann-Whitney test, *p*-value = 0.0043) (Fig. 5c).

We then performed optical super-resolution imaging of fluorescently-labelled early endosomes using Super-Resolution Structured Illumination Microscopy (SR-SIM) with a lateral resolution of 100 nm (Fig. 5d). Morphometric analysis revealed that the volume of EEA1-positive puncta was not significantly different between euploid (*n* = 11 cells, mean volume = 0.017 μm^3^, SEM ± 0.0012) and T21 isogenic neurons (*n* = 14 cells, mean volume = 0.015 μm^3^, SEM ± 0.0009) (mixed effects ANOVA, genotype *p*-value = 0.95) (Fig. 5e). We counted the number of clusters of EEA1-positive puncta and found a 1.3-fold increase in T21 neurons (*n* = 14 cells, mean number of clusters/total number = 0.064, SEM ± 0.0054) when compared to euploid isogenic neurons (*n* = 10 cells, mean number of clusters/total number = 0.049, SEM ± 0.0036) (Mann-Whitney test, *p*-value = 0.047) (Fig. 5f).

### Gene expression profiling in fibroblasts from individuals with DS using RNAseq

Based on the strong morphological alterations of the endosomal compartment identified in DS, we hypothesized that members of the endosomal machinery could be deregulated in DS. In order to address this hypothesis, we analyze gene expression using RNA sequencing in 3 euploid and 6 DS fibroblast lines (Supplementary Table). Data analysis revealed that 26,423 genes were expressed in at least one sample. We found 1073 differentially expressed (DE) genes in DS fibroblasts as compared to 2 N using DESeq2 and a false discovery rate of 5%, with a significant enrichment of genes mapping to HSA21 (adjusted *p*-value = 2.9538.10^− 16^). No significant enrichment was found for other chromosomes. The entire list of genes is available in Online Resource 1.

**Fig. 6:**
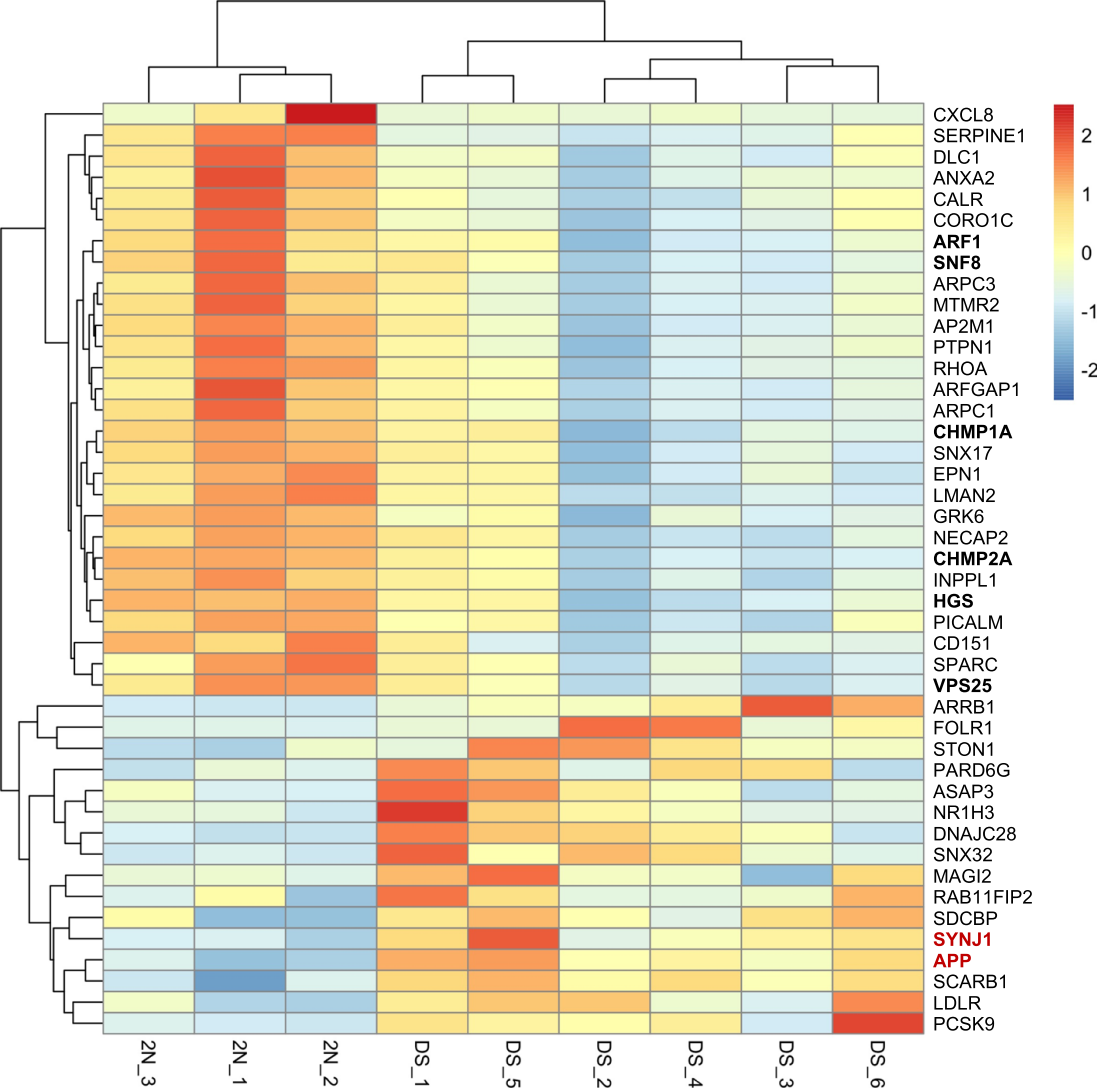
Heatmap of the 44 genes related to endocytosis that were differentially expressed genes between DS and euploid fibroblasts. Each column represents a cell line, each row a specific probe. Red indicates relative gene upregulation, and blue indicates relative gene downregulation. Hierarchical clustering classified DS subjects together and separated from euploid (2 N), showing a differential expression pattern between the groups. *APP* and *SYNJ1* genes are highlighted in red bold, while differentially expressed genes related to cargo sorting from early endosomes to multivesicular bodies (MVB) toward the degradation pathway are highlight in black bold

Among the 1084 DE genes, 503 genes were downregulated (log2 fold change from − 0.18 to − 7.53) while 581 genes were overexpressed (log2 fold change from 0.29 to 7.05). From this list of 1084 DE genes, we selected 44 genes related to endocytosis and to the endosomal pathway based on their GO and KEGG entries. Hierarchical clustering showed that these 44 DE genes were able to differentiate between 2 N and DS conditions. Heat map revealed two clusters of DE genes, one with 28 genes dowregulated in DS and the other with 16 genes overexpressed in DS (Fig. 6). Interestingly, we observed that 6 genes belonging to the first cluster were related to cargo sorting from early endosomes to multivesicular bodies (MVB) toward the degradation pathway (*ARF1*, *HGS*, *CHMP1A*, *CHMP2A*, *SNF8* and *VPS25*).

### MVB-dependent degradation pathway in fibroblasts from individuals with DS

**Fig. 7:**
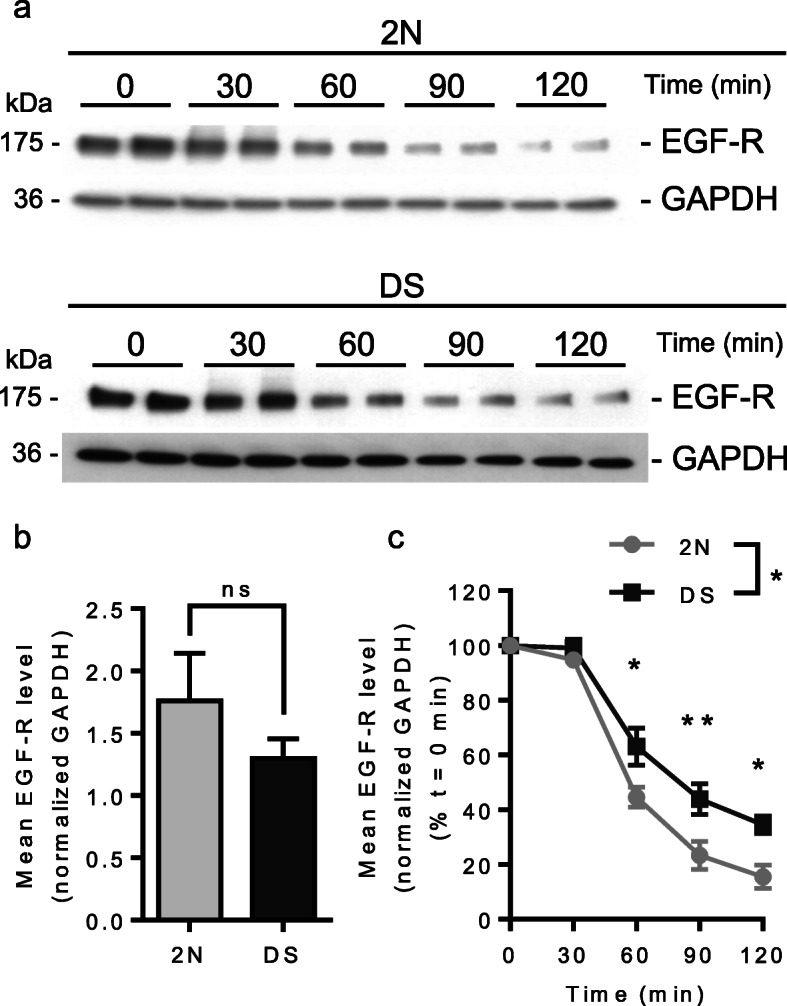
MVB-dependent degradation of EGF-R in fibroblasts from individuals with DS and euploid controls. **a** Western blot of EGF-R in euploid fibroblasts and fibroblasts from individuals with DS illustrating the degradation of the EGF-R at times 0, 30, 60, 90 and 120 min of EGF treatment. GAPDH is used for normalization; **b** Mean EGF-R level at time = 0 min is not significantly different between euploid (*n* = 3 individuals) and DS fibroblasts (*n* = 3 individuals) (three independent experiments each including one euploid and one DS individual) (Mann-Whitney test, *p*-value = 0.4); **c** Quantification of EGF-R level expressed as a percentage of EGF-R level at time = 0 min in each condition and normalized to GAPDH level shows that the degradation of EGF-R is significantly delayed in fibroblasts from individuals with DS as compared to euploid fibroblasts (Two-way ANOVA, *p-value = 0.012; *post-hoc* Bonferroni test, adjusted *p*-value at t_30 min_ > 0.99, *adjusted *p*-value at t_60 min_ = 0.018; **adjusted *p*-value at t_90 min_ = 0.0077, *adjusted *p*-value at t_120 min_ = 0.015)

Based on the RNAseq analysis showing that a number of genes related to cargo trafficking in the degradation pathway are downregulated in fibroblasts from individuals with DS, we assessed MVB-dependent degradation pathway by measuring the degradation of EGF receptors (EGF-R). EGF-R internalization and degradation is induced by binding of EGF [51]. We thus treated 2 N and DS fibroblasts with EGF (50 ng/mL) for up to 2 h and analyzed EGF-R degradation by western blot. Figure 7a shows that EGF-R level decreased over time both in 2 N and DS fibroblasts. At time 0, the level of EGF-R was not significantly different between 2 N (*n* = 3 individuals, EGF-R/GAPDH level = 1.76, SEM ± 0.38) and DS fibroblasts (*n* = 3 individuals, EGF-R/GAPDH level = 1.298, SEM ± 0.16) (Mann-Whitney test, *p*-value = 0.4) (Fig. 7b). Comparison of EGF-R degradation curve revealed a significant delay in DS fibroblasts when compared to 2 N (Two-way ANOVA, genotype *p*-value = 0.012). *Post-hoc* analysis between genotypes for each time point showed significant difference at 60, 90 and 120 min following EGF treatment (*post-hoc* Bonferroni test, adjusted *p*-value at t_30 min_ > 0.99, adjusted *p*-value at t_60 min_ = 0.018; adjusted *p*-value at t_90 min_ = 0.0077, adjusted *p*-value at t_120 min_ = 0.015) (Fig. 7c). These results show that MVB-dependent degradation of EGF-R is delayed in DS fibroblasts.

### Endocytosis and recycling of the transferrin receptors in fibroblasts from individuals with DS

**Fig. 8:**
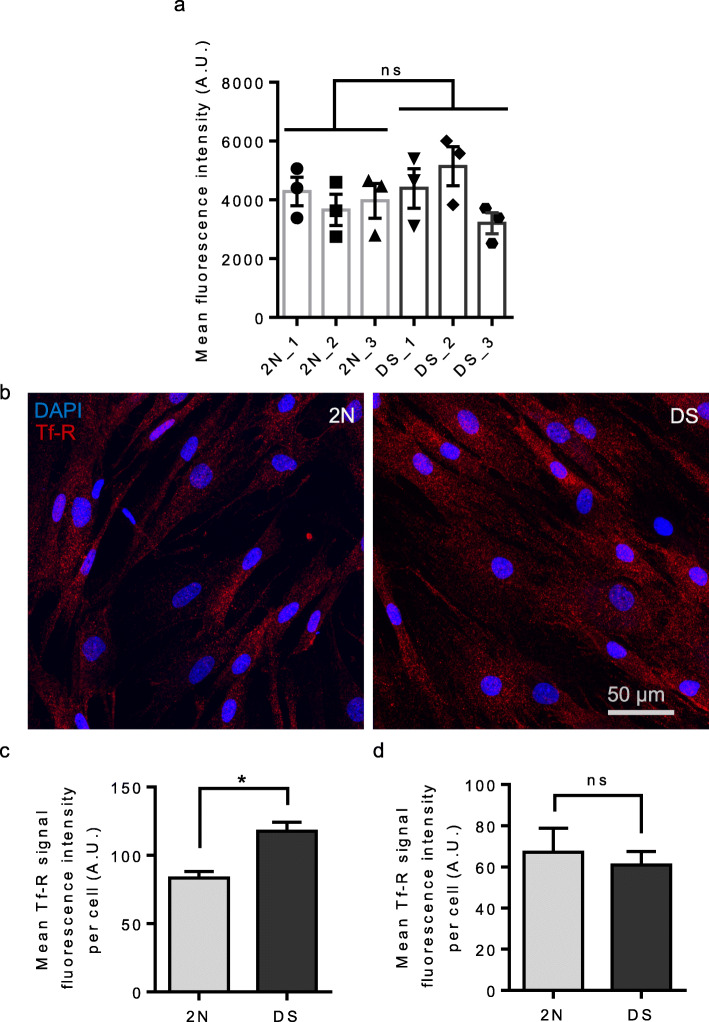
Endocytosis and recycling of transferrin receptors in fibroblasts from individuals with DS and euploid controls. **a** Quantification of 4 min endocytosis of transferrin-Alexa Fluor 647 in fibroblasts from 3 euploid individuals and 3 individuals with DS. Mean fluorescence intensity of transferrin is not significantly different between 2 N fibroblasts (*n* = 3 individuals, mean fluorescence intensity = 3970, SEM ± 283.3) and fibroblasts from individuals with DS (*n* = 3 individuals, mean fluorescence intensity = 4248, SEM ± 405.5) (three independent experiments each including all individuals) (Mann-Whitney test, *p*-value = 0.53); **b** Representative z-projected stack images of transferrin receptors recycled to the cell surface (red) and DAPI (blue) in euploid fibroblasts and fibroblasts from individuals with DS; **c** Quantification of fluorescence intensity of the transferrin receptor surface staining after 15 min of transferrin recycling shows a significant increase in the mean fluorescence intensity in DS fibroblasts as compared to euploid fibroblasts (mixed effects ANOVA, genotype *p-value = 0.012) (two independent experiments each including all individuals); **d** Quantification of fluorescence intensity of the transferrin receptor surface staining in cultured untreated fibroblasts from euploid fibroblasts and fibroblasts from individuals with DS shows no significant difference between genotypes (mixed effects ANOVA, genotype *p*-value = 0.67)

After showing that the MVB-dependent degradation pathway is impaired in DS fibroblasts, we analyzed the dynamic of endocytosis and endosomal recycling. First, we studied endocytosis using a conventional method for measuring the levels of internalized fluorescent transferrin by flow cytometry [49, 65]. We treated 3 2 N and 3 DS fibroblasts with Alexa647-transferrin for 4 min at 37 °C, fixed the cells and analyzed intracellular fluorescence levels of internalized transferrin and repeated the experiments three times. We could not identify significant differences in the level of transferrin internalization between 2 N and DS fibroblasts (Mann-Whitney test, *p*-value = 0.53) (Fig. 8a).

We next studied the recycling of the transferrin receptor. Fibroblasts kept at 4 °C to block endocytosis were treated with transferrin for 15 min at 37 °C to induce internalization and recycling of the transferrin receptors, and then washed and fixed. Transferrin receptors recycled to the cell membrane were immunostained without permeabilization using specific antibody (Fig. 8b). We found a significant increase in fluorescence intensity per cell in DS fibroblasts (*n* = 3 individuals, mean fluorescence intensity = 117.5, SEM ± 6.85) when compared to 2 N fibroblasts (*n* = 3 individuals, mean fluorescence intensity = 83.35, SEM ± 4.85) (mixed effects ANOVA, genotype *p*-value = 0.012) (Fig. 8c). This increase of transferrin receptor recycling was not due to higher transferrin receptor density in cultured untreated DS fibroblasts as compared to 2 N measured by fluorescent immunostaining (*n* = 3 individuals, mean fluorescence intensity = 67.12, SEM ± 11.63) and DS fibroblasts (*n* = 3 individuals, mean fluorescence intensity = 60.95, SEM ± 6.49) (mixed effects ANOVA, genotype *p*-value = 0.67) (Fig. 8d).

Altogether our data suggest that in DS condition transferrin receptor endocytosis is not consistently modified while its recycling is upregulated.

### PI (3) P levels in fibroblasts from individuals with DS

**Fig. 9:**
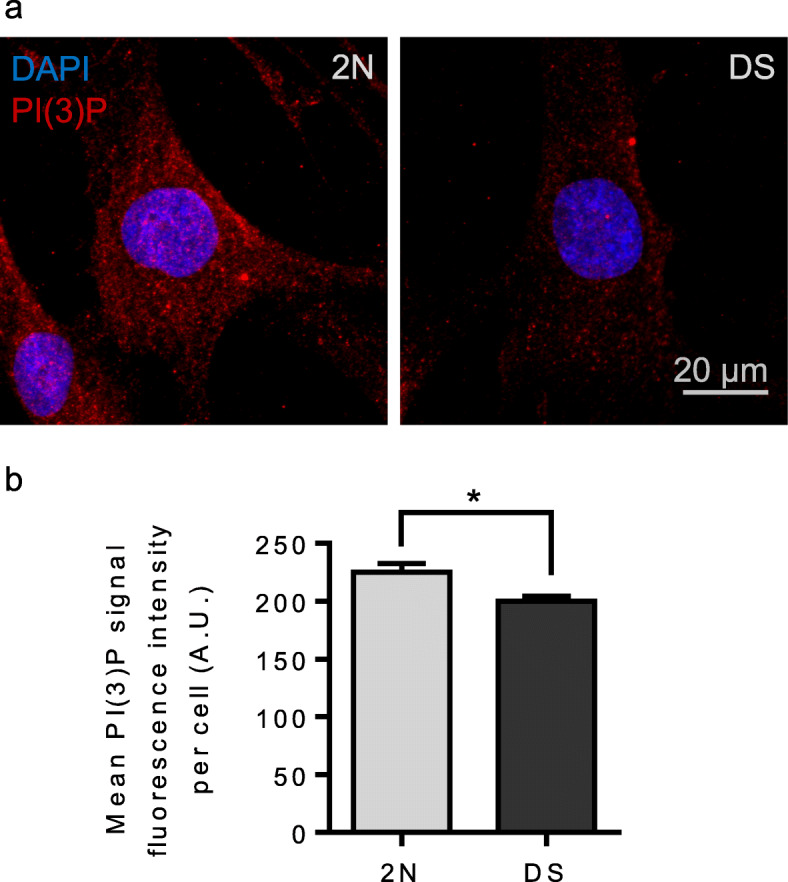
PI (3) P level in fibroblasts from individuals with DS and euploid controls. **a** Representative images of PI (3) P staining (red) and DAPI (blue) in euploid fibroblasts and fibroblasts from individuals with DS; **b** Quantification of the PI (3) P signal fluorescence intensity shows a significant decrease in the mean PI (3) P fluorescence intensity in fibroblasts from individuals with DS as compared to euploid fibroblasts (mixed effects ANOVA, genotype *p-value = 0.043)

Phosphoinositides play a major role in intracellular trafficking. Early endosomes are particularly enriched in PI (3) P, at the crossroad of various endosomal pathways [27]. The levels of PI (3) P are significantly decreased in the brain of AD patients and in mouse models of FAD [58]. Considering the delay in EGF-R degradation and the increase in recycling of transferrin receptors in fibroblasts from individuals with DS, we suspected that PI (3) P levels could be deregulated in DS. We immunostained PI (3) P in 2 N and DS fibroblasts with a specific anti-PI (3) P antibody [28] and measured the mean fluorescence intensity per cell in 2 N and DS fibroblasts (Fig. 9a). We found a significant decrease in PI (3) P fluorescence intensity in DS fibroblasts (*n* = 3 individuals, mean fluorescence intensity = 199.9, SEM ± 4.28) as compared to 2 N fibroblasts (*n* = 3 individuals, mean fluorescence intensity = 225.2, SEM ± 7.33) (mixed effects ANOVA, genotype *p*-value = 0.043) (Fig. 9b). As predicted, our experiments indicate that the levels of PI (3) P are decreased in DS fibroblasts as compared to 2 N.

## Discussion

Enlargement of early endosomes in DS and AD has been mainly characterized over the past two decades using conventional light microscopy at a resolution of around 200 nm, corresponding to the actual size of these subcellular organelles [9–14, 19–21, 68]. In order to overtake the diffraction limit, we used super-resolution and transmission electron microscopies to analyze the early endosomal compartment in DS condition and compared to conventional confocal fluorescent microscopy. Moreover, we relied on a method enabling observations of optimally preserved structures by EM using HPF of live cells. We accessed four cell types: LCLs and fibroblasts from individuals with DS, human neurons derived from T21 and 2 N isogenic iPSC clones from an individual with a mosaic T21 and neuronal cells from the brain of Ts65Dn mice modelling DS.

### Early endosomes morphology in DS studied at high-resolution

We first analyzed the morphology of early endosomes by immunofluorescence using confocal microscopy. In fibroblasts from individuals with DS, we found an 18% increase in volume of EEA1-positive puncta, in line with previous studies in DS fibroblasts, LCLs and peripheral mononuclear blood cells [11, 20]. We next analyzed for the first time early endosome morphology in human T21 and 2 N isogenic neurons derived from iPSC clones obtained from an individual with mosaicism for T21. Using confocal microscopy, EEA1-positive puncta increased by 13% in T21 human neurons as compared to 2 N, which is consistent with a previous study in iPSC-derived neurons from individuals with sporadic AD [44]. Our study validates the use of this cellular model for the study endosomal dysfunctions in DS. Additionally, the study of isogenic cells is valuable in regard of inter-individual variability. Overall, despite variability between cell types, confocal microscopy revealed a significant enlargement of EEA1-positive puncta.

In order to visualize early endosomes at high resolution, we performed SR-SIM on 2 N and T21 human isogenic neurons derived from iPSC clones. SR-SIM allows 3 dimensional imaging, otherwise technically limited by EM. By SR-SIM, EEA1-positive puncta volume was unchanged between euploid and DS condition. We counted the number of clustered puncta (i.e. individual EEA1-positive puncta close by) and found significantly more clusters in T21 neurons (increase of 31%).

Defining the morphology of early endosomes based on endosomal protein fluorescence staining appears arduous, as EEA1 domains are hardly distinguishable from endosomes by SR-SIM. Indeed, proteins used to identify early endosomes (e.g. Rab5 and EEA1) are usually not uniformly distributed at the surface of early endosomes, as early endosomal molecular machinery is sequentially recruited on functional microdomains at the endosomal membrane [33, 64]. Thus, the observation of endosomal proteins by fluorescence staining using confocal microscopy can only reveal puncta-like signals, corresponding to protein microdomains at the endosomal surface. This aspect is critical, because it implies that confocal microscopy observations rather relate to the morphology of endosomal protein microdomains rather than to the global early endosomal morphology, especially when early endosomes are large enough for microdomains to be visible [33, 57, 58, 63]. Experiments resulting in the enlargement of early endosomes, achieved via transfection of Rab5-Q79L, clearly show the clusters of high intensity of endosomal proteins, such as EEA1, at the endosomal limiting membrane [33, 55, 63, 64]. Likewise, the protein microdomains can be observed when endosomes are imaged using super-resolution microscopy [30]. Nevertheless, it is unlikely that the clusters of EEA1-positive puncta shown in the insets of Fig. 5d correspond to one single endosome, as that endosome would then need to have a diameter > 1.5 μm, which is three times higher than the diameter determined by confocal microscopy (449 nm for a mean volume of 0.0477 μm^3^). Hence, this lead us to the conclusion that quantifications of immunofluorescence staining of early endosome markers such as EEA1 or Rab5 observed by confocal microscopy and super-resolution microscopy are not sufficient to characterize the early endosome morphology.

Electron microscopy provided the solution to the aforementioned limitations of the optical microscopy. Firstly, we studied the morphology of the EEA1-positive early endosomes in LCLs and fibroblasts from individuals with DS by EM after aldehyde fixation. In LCLs, the morphometric analysis showed that the early endosomal area is unchanged between euploid and DS LCLs. Early endosomes were often clustered, without apparent attachment between them. In fibroblasts from individuals with DS, EM revealed similar clusters of EEA1-positive early endosomes. In order to prevent any artefacts due to chemical fixation, we analyzed the morphology of early endosomes by electron microscopy after HPF in 2 N and DS fibroblasts. HPF induces a fast vitrification of live cells, which allows for the preservation of morphology of intracellular components, including early endosomes [24, 29, 59]. Using this technique, we found that the mean early endosome size in DS fibroblasts was unchanged, similarly to early endosomes in DS LCLs visualized by EM after chemical fixation. Early endosomes were significantly more numerous in DS fibroblasts as compared to 2 N fibroblasts (density increased by 75%). The clustering phenomenon observed by EM after chemical fixation in DS LCLs and fibroblasts was very rare after HPF, indicating that it could be an artefact due to increased density of endosomes, and to the use of fixative that has been shown to favor the clustering of synaptic vesicles [48, 66].

Overall, we explored endosomal dysfunctions in DS fibroblasts, in human isogenic neurons derived from iPSC and in the brain of mice modelling DS. It should be noticed that our collection of euploid and DS fibroblasts was not equilibrated for age. However, previous publication showed that age did not interfere with the endosome morphology of neurons in control subjects [14]. It will be interesting to confirm our results using high-resolution live imaging on neuronal cells in culture and super resolution immunohistochemistry or structural EM on human post-mortem tissue.

### Defective cargo trafficking in DS fibroblasts

We next chose to extend the characterization of the endosomal pathway in our collection of fibroblasts as the increased number of early endosomes observed by EM after HPF in DS fibroblasts suggested alterations in the dynamic routes of the endosomal pathway. RNA sequencing of fibroblasts from individuals with DS and controls revealed that the expression pattern of differentially expressed genes related to endocytosis and endosomal pathways was distinct between 2 N and DS fibroblasts. Among these genes, a sub-group is specifically related to cargo sorting at MVB via ESCRT (Endosomal Sorting Complexes Required for Transport) regulation. ARF1 was associated with MVB formation through pH-dependent recruitment of coat proteins complexes [35]. HGS, also known as Hrs, is a well-characterized effector of PI (3) P which is recruited to the endosome membrane by binding of its FYVE domain to PI (3) P [63]. It carries an interaction motif with ubiquitin and associates with the protein STAM (Signal Transducing Adapter Molecule 1), thus forming the ESCRT-0 (endosomal sorting complex required for transport) complex which sorts ubiquitinylated cargo toward the degradation pathway via MVB [2, 3]. SNF8 also known as Vps22, and Vps25 are members of ESCRT-II implicated in membrane budding [41]. CHMP1A and CHMP2A (charged multivesicular body protein 1A and 2A) are members of the ESCRT-III protein complex that participate to Vps4 recruitment, enabling ESCRT-III disassembly after intraluminal vesicle formation [56]. Overall, deregulations of genes related to ESCRT machinery point toward defective MVB formation and cargo sorting for degradation.

In AD context, APP overexpression and Aβ accumulation were found to disrupt EGF-R degradation in APP transgenic mice carrying the Swedish mutation, by inhibiting the UPS (Ubiquitin Proteasome System) [1]. As the APP gene is located on HSA21 and is overexpressed in DS, we could suspect that a similar mechanism occurs in DS context. In line with this data, we identified that the MVB-dependent degradation of EGF-R is delayed in fibroblasts from individuals with DS. This result, in addition to the increased number of early endosomes and the altered expression of ESCRT-related genes in DS fibroblasts, strongly implies defective cargo sorting and traffic jam at early endosomes in DS fibroblasts. Mutant APP showing altered sorting to MVB leads to increased Aβ production in neurons [58]. Thus, defective MVB sorting could in turn play a central role in APP trafficking and Aβ production. We also showed that the recycling of the transferrin receptor is increased in DS fibroblasts. This result is consistent with previous studies showing that Rab4-positive vesicles involved in the recycling pathway are more numerous and Rab4 expression is increased in DS fibroblasts [11, 78] and in the Ts65Dn mouse model of DS [12]. In DS, overexpression of the miRNA-155 mapping to HSA21 negatively regulates the transcription of SNX27 [81]. As SNX27 is a member of the retromer machinery, we can suspect that the retromer-dependent sorting of cargos toward the recycling pathway is impaired in DS. Overall, our results unveil a global deregulation of the dynamic routes emanating from early endosomes thus creating a traffic jam already proposed in the context of AD [74].

### Molecular bases of endosomal dysfunction in DS

APP overexpression in DS, confirmed in our collection of DS fibroblasts by RNAseq, is involved in endosomal defects observed in DS. Indeed, several lines of evidence suggest that higher APP gene dosage causing increased expression of β-CTF could be responsible for early endosome enlargement in DS [45]. Higher levels of β-CTF would stimulate APPL1 recruitment to early endosomes thereby stabilizing the GTP-bound form of Rab5 and promoting endosomal fusion [47]. Additionally, abnormal Rab5 activation found in Ts65Dn mice, due to increased APP and β-CTF expression, was shown to disrupt NGF axonal transport involved in BFCNs degeneration [86]. However, EM imaging of giant early endosomes induced by Rab5-GTP overexpression [82] shows a clearly distinct morphology as compared to the ultrastructure of early endosomes observed by EM in DS in the present study.

Other hypotheses can be formulated to explain the role of overexpressed genes in endosomal dysfunction in DS. We previously demonstrated that HSA21 gene synaptojanin1 (*SYNJ1*), overexpressed in DS, is involved in early endosome alterations in DS [20]. Down-regulation of SYNJ1 reversed endosomal enlargement in fibroblasts from individuals with DS [20], improved Aβ clearance and memory deficits in Ts65Dn mice [80] and in AD mouse model [87]. SYNJ1 is involved in phosphoinositides metabolism, mostly acting as a 5-phosphatase. Among the seven members of the phosphoinositides family of phospholipids, PI (3) P, PI (4) P, PI (4,5) P_2_ and PI (3,4,5) P_3_ are associated with AD. PI (3) P has a major role in early endosome fusion since dysregulation of PI (3) P hinders fusion between vesicles. The phosphatidylinositol-3 kinase (PI (3)-kinase) Vps34 phosphorylates PI to PI (3) P and is activated by Rab5-GTP, thereby promoting the assembly of PI (3) P with Rab5 and Rab5 effectors via FYVE motives [17, 31, 61, 72, 75]. PI (3) P levels are decreased in the brain of patients with sporadic AD and in mice models of AD, and silencing the PI (3)-kinase Vsp34 leads to APP processing dysfunction in endosomes [58]. We found that PI (3) P level is decreased in fibroblasts from individuals with DS, in accordance with AD context. In light of recent findings showing that APP binds PIKfyve, the kinase that phosphorylates PI (3) P to PI (3,5) P_2_ [5, 22, 23], APP and phosphoinositides interaction can be suspected to cause endosome traffic jam. Indeed APP binding to PIKfyve leads to an over-production of PI (3,5) P_2_, which has been shown to negatively regulate fusion between early endosomes [42, 43, 69]. In DS, APP over-expression could promote PIKfyve activation and thereby lower PI (3) P level. Fusion deficits caused by a lack of PI (3) P would make early endosomes prone to clustering. Together our data reveals PI (3) P deregulation, defective cargos sorting to the degradation pathway and increased recycling of the transferrin receptor in DS fibroblasts. In turn, APP overexpression and Aβ overproduction could participate to these defects.

## Conclusions

Our work highlights the significance of early endosome dysfunction in DS and redefines early endosome phenotype in DS which remained ignored due to the lack of resolution of conventional light microscopy. Using the resolving power of SR-SIM and EM, suited to the study of the endosomal compartment, we could show that in DS fibroblasts early endosomes are normal-sized but, in the presence of fixatives, they tend to aggregate more than in the control situation supposedly because they are more numerous. These results question the use of confocal microscopy for the description of subcellular compartments such as endosomes in disease conditions. Although EM imaging of *postmortem* human brain sections remains a challenge, the use of SR-SIM might be an alternative to reach super resolution. Combined ultrastructure and dynamics of endocytosis, recycling and degradation in DS fibroblasts point to the endosomal “traffic jam” hypothesis recently formulated in AD [74]. The complexity of endosomal regulation and the high number of genes triplicated in DS suggest multifactorial causes for endosomal abnormalities. Here we unveil new mechanisms involving phosphoinositides such as PI (3) P, an identified target in sporadic AD.

## Supplementary information

**Additional file 1: Supplentary Figure S1.** Representative image of a cluster of EEA1-DAB labelled endosomes in a LCL cell from an individual with DS. Electron micrographs along 8 serial 50 nm-thick sections. Note that sections 3 and 6 are missing. Individual endosomes are identified by differently colored dots, and most of them can be seen in 2 successive sections. The cluster contains at least 17 distinct endosomes and would appear as polycyclic if it was fluorescently labelled. Note that the DAB precipitate accumulating around surrounding small vesicles makes them appear as pseudo-coated.

**Additional file 2: Supplementary Table.** Demographic description of fibroblasts from euploid individuals and individuals with DS.

**Additional file 3.**

## Data Availability

The datasets used and/or analyzed during the current study are available from the corresponding author on reasonable request.

## Abbreviations

AD: Alzheimer’s disease
ANOVA: Analysis of variance
APOE: Apolipoprotein E
APP: Amyloid Precursor Protein
APPL: Adaptor Protein, Phosphotyrosine interacting with PH domain and Leucine zipper
Aβ: Amyloid β
BFCN: Basal forebrain cholinergic neurons
DE: Differentially expressed
ChAT: Choline acetyltransferase
CTF: C-terminal fragment
DAB: 3′-Diaminobenzidine
DNA: Deoxyribonucleic AcidDS Down Syndrome
EEA1: Early Endosome Antigen 1
EGF: Epidermal growth factor
EM: Electron microscopy
ESCRT: Endosomal sorting complex required for transport
FAD: Familial Alzheimer’s disease
FISH: Fluorescence in situ hybridization
GAPDH: Glyceraldehyde 3-phosphate dehydrogenase
GTP: Guanosine Triphosphate
GWAS: Genome Wide Association Study
HPF: High-pressure freezing
HRP: Horseradish peroxidase
HSA: Human chromosome
ID: Identifier
iPSC: Induced pluripotent stem cells
LCL: Lymphoblastoid cell line
MSN: Medial Septum Nucleus
MVB: Multivesicular body
NCS: Neural stem cells
PBMC: Peripheral blood mononuclear cells
PCR: Polymerase Chain Reaction
PI(3)P: Phosphatidylinositol 3 Phosphate
RNAseq: Ribonucleic Acid sequencing
ROI: Region Of Interest
SEM: Standard Error of the Mean
SR-SIM: Super-Resolution Structured Illumination Microscopy
SYNJ1: Synaptojanin-1
TEM: Transmission electron microscopy
T21: Trisomy 21
WT: Wild-Type
2 N: Euploid
3D: 3 Dimensional
3 N: Triploid
