# Supplementary figures and images for: Ultrastructural and dynamic studies of the endosomal compartment in Down syndrome
Source: Acta Neuropathol Commun. 2020 Jun 24;8:89. doi: 10.1186/s40478-020-00956-z (PMC7315513; doi:10.1186/s40478-020-00956-z)

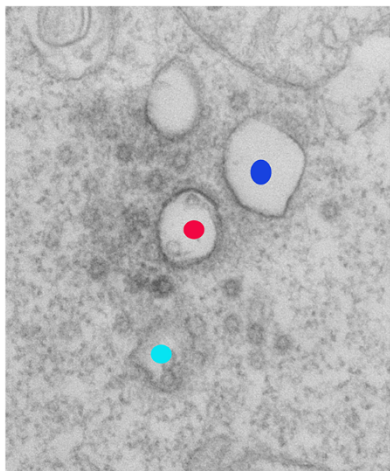

Section 1

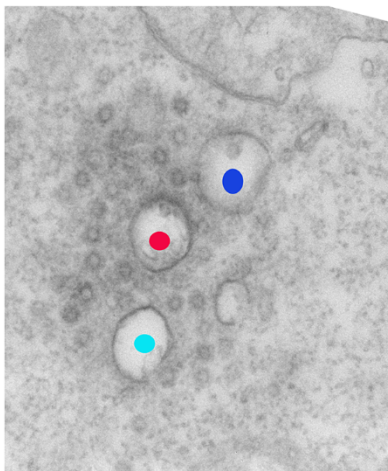

Section 2

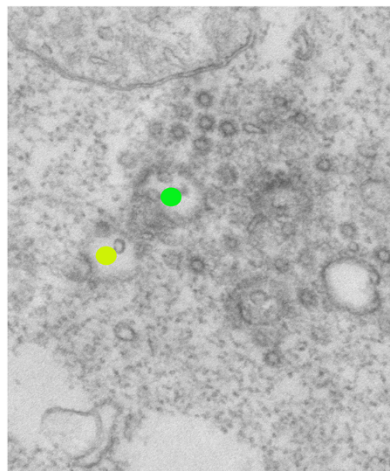

Section 4

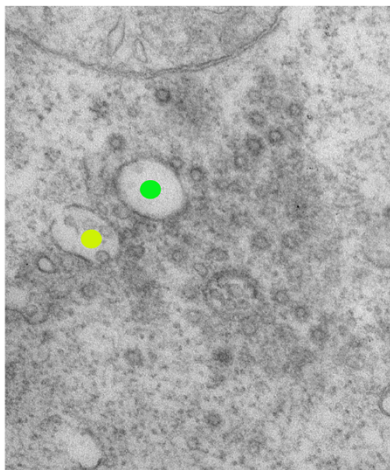

Section 5

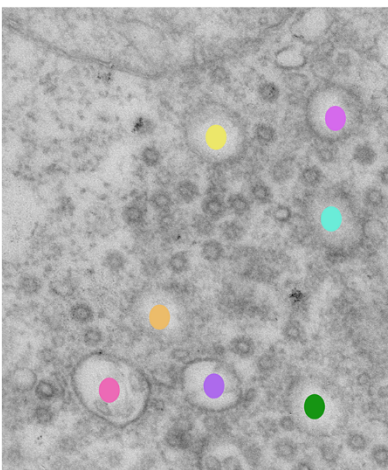

Section 7

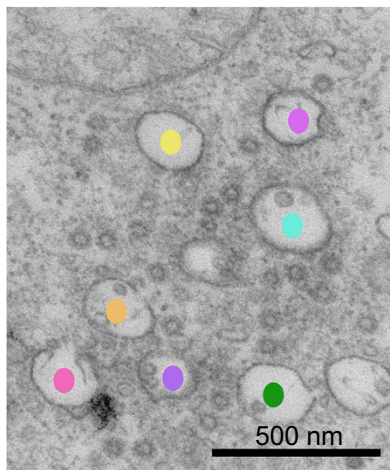

Section 8

Supplement: Supplementary file 1 — Additional file 1: Supplentary Figure S1. Representative image of a cluster of EEA1-DAB labelled endosomes in a LCL cell from an individual with DS. Electron micrographs along 8 serial 50 nm-thick sections. Note that sections 3 and 6 are missing. Individual endosomes are identified by differently colored dots, and most of them can be seen in 2 successive sections. The cluster contains at least 17 distinct endosomes and would appear as polycyclic if it was fluorescently labelled. Note that the DAB precipitate accumulating around surrounding small vesicles makes them appear as pseudo-coated. [file 40478_2020_956_MOESM1_ESM.pdf]
