# Supplementary material for: Ultrastructural and dynamic studies of the endosomal compartment in Down syndrome
Source: Acta Neuropathol Commun. 2020 Jun 24;8:89. doi: 10.1186/s40478-020-00956-z (PMC7315513; doi:10.1186/s40478-020-00956-z)
Supplement: Supplementary file 2 — Additional file 2: Supplementary Table. Demographic description of fibroblasts from euploid individuals and individuals with DS. [file 40478_2020_956_MOESM2_ESM.docx]

| **Line number** | **Line ID** | **Supplier** | **Karyotype** | **Age at sampling** | **Gender** |
| --- | --- | --- | --- | --- | --- |
| 2N_1 | GM05659 | Coriell Institute | 2N | 1 | M |
| 2N_2 | 94 | ImaBio3 cohort | 2N | 66 | M |
| 2N_3 | 69 | ImaBio3 cohort | 2N | 69 | F |
| DS_1 | AG05397 | Coriell Institute | 3N | 1 | M |
| DS_2 | TOM | Institut Jérôme Lejeune | 3N | 21 | M |
| DS_3 | FRA | Institut Jérôme Lejeune | 3N | 36 | F |
| DS_4 | GUC | Institut Jérôme Lejeune | 3N | 37 | F |
| DS_5 | SAA | Institut Jérôme Lejeune | 3N | 34 | M |
| DS_6 | BAQ | Institut Jérôme Lejeune | 3N | 26 | M |
